# Bromodomain factors of BET family are new essential actors of pericentric heterochromatin transcriptional activation in response to heat shock

**DOI:** 10.1038/s41598-017-05343-8

**Published:** 2017-07-14

**Authors:** Edwige Col, Neda Hoghoughi, Solenne Dufour, Jessica Penin, Sivan Koskas, Virginie Faure, Maria Ouzounova, Hector Hernandez-Vargash, Nicolas Reynoird, Sylvain Daujat, Eric Folco, Marc Vigneron, Robert Schneider, André Verdel, Saadi Khochbin, Zdenko Herceg, Cécile Caron, Claire Vourc’h

**Affiliations:** 1 Université Grenoble Alpes, CNRS UMR 5309, INSERM U1209, Institute for Advanced Biosciences (IAB), Site Santé - Allée des Alpes, 38700 La Tronche, France; 2 International Agency for Research on Cancer (IARC), 69008 Lyon, France; 3 UMR 7242, Ecole Supérieure de Biotechnologie de Strasbourg (ESBS), 300 boulevard Sebastien Brant, CS 10413, 67412 Illkirch, France; 4 Institute of Genetics and Molecular and Cellular Biology (IGBMC), Strasbourg, France; 5 Institute of Functional Epigenetics, Helmholtz Zentrum Muenchen, Ingolstaedter Landstr 1, 85754 Neuherberg, Germany

## Abstract

The heat shock response is characterized by the transcriptional activation of both hsp genes and noncoding and repeated satellite III DNA sequences located at pericentric heterochromatin. Both events are under the control of Heat Shock Factor I (HSF1). Here we show that under heat shock, HSF1 recruits major cellular acetyltransferases, GCN5, TIP60 and p300 to pericentric heterochromatin leading to a targeted hyperacetylation of pericentric chromatin. Redistribution of histone acetylation toward pericentric region in turn directs the recruitment of Bromodomain and Extra-Terminal (BET) proteins BRD2, BRD3, BRD4, which are required for satellite III transcription by RNAP II. Altogether we uncover here a critical role for HSF1 in stressed cells relying on the restricted use of histone acetylation signaling over pericentric heterochromatin (HC).

## Introduction

In human cells, the heat shock response (HSR) is characterized by the transcriptional activation of both protein coding heat shock (hsp) genes^1^ and pericentric heterochromatin (HC) formed of tandem repeats of sat III elements over several megabasis^2, 3^. Both events are under the control of Heat Shock Factor 1 (HSF1), a transcription factor present in the nucleus and cytoplasm of cell before heat shock^4^. Activation of pericentric HC is mainly observed at the 9q12 locus, and leads to the accumulation of long noncoding (lnc) satellite III (sat III) RNA. Sites of HSF1 accumulation at pericentric HC and of sat III sequences transcription form nuclear foci, also called nuclear stress bodies, (nSBs) in stressed cells. Transcriptional regulation of the heat shock response at hsp gene promoters, has been extensively studied as a model system of gene expression. Unlike hsp genes, sat III transcripts are transcribed from heterochromatic regions suggesting that different mechanisms may be involved in their transcriptional activation. In higher eukaryotes, RNA pol II (RNAP II) at hsp genes is transcriptionally engaged^5^ before heat shock, while no evidence exist that it is similarly engaged at sat III sequences.

Several actors have been identified for their role in HSF1-mediated hsp genes activation in higher eukaryotes. In Drosophila, proteins have been identified for their essential role in the initial, HSF1-mediated, transcription independent, nucleosome disruption. They include GAGA factor (GAF; Trithorax-like) and poly(ADP)-ribose polymerase (PARP)^6, 7^. TIP60 has also been identified for its role in the spreading of PARP and subsequent recruitment of elongation factors such as P-TEFb and Spt6 and for its role in the recruitment of histone-modifying enzymes such as Trithorax and CREB-binding protein (CBP)^6–8^. Based on *in vitro* interaction assays, a binding between HSF1, the general transcription factors TATA-Binding Protein (TBP) and Transcription Factor IIB (TFIIB) has been reported in human cell extracts^9^. More recently a direct interaction between HSF1 and replication protein A (RPA), which is known to bind and stabilize single-strand DNA (ssDNA) has also been characterized^10^. The HSF1-RPA complex leads to preloading of RNAP II and opens the chromatin structure by recruiting a histone chaperone, FACT^10^.

Histone modifications, and especially acetylation, are known to play an essential role in the control of gene regulation^11^. The role of Histone Acetyl Transferases (HATs) is to catalyze lysine acetylation which, depending on the lysine targeted, could be a way to loosen histone/DNA interactions^12^. Acetylated residues also represent docking sites for bromodomain (Brd), a protein domain known to act as an acetyl-lysine binding domain. Brd-containing factors include proteins with multiple roles in transcriptional activation, like the HATs GCN5, PCAF (p300/CREB-binding protein-Associated Factor), p300 and like CBP (CREB Binding Protein), the BRG1 ATPase subunit of SWI/SNF nucleosome remodeling complex, or the TAF1 subunit of the general transcription factor TFIID (Transcription initiation factor TFIID subunit 1). They also include the “bromo- and extra terminal domains” (BET) family members, among which BRD2, BRD3, BRD4 and BRDT^13^. BRD4 and BRDT are key mediators for the assembly of the Positive Transcription Elongation Factor b complex (P-TEFb), an event required for the initiation of transcription elongation^14, 15^. An intrinsic histone chaperone activity has also been characterized for BRD2^16^. BET/histone interactions, which play important roles in physiological^15, 17^ and in pathological settings^18^, can be selectively inhibited by the acetyl-lysine competitive inhibitor such as JQ1^19, 20^. As expected, histone acetylation plays an important role in the activation of hsp genes. Likewise, increased acetylation at histone H3 in the presence of HSF1 at the hsp70 gene is associated with increased recruitment of CBP and BRG1^21^, while an interaction between HSF1, p300/CBP and STRAP (Stress-responsive activator of p300) promotes HSF1 binding and augments histone acetylation within hsp genes^22^. Important changes in chromatin acetylation have also been shown within nSBs, revealed by the appearance of HSF1-dependent acetylated foci at nSBs, colocalizing with foci enriched for the HAT p300/CBP^2^. However the role of the different actors described above, that set up and read acetylation marks, is currently unknown in the transcriptional activation of sat III sequences. The contrasting role of Trichostatin A (TSA), a potent inhibitor of Histone Deacetylases of Class I and II mammalian Histone Deacetylases (HDACs), on sat III and hsp70 transcriptional activation supports the existence of distinct specificities between sat III and hsp gene transcription. Indeed, while TSA promotes hsp 70 gene transcription even in the absence of stress, TSA inhibits sat III transcriptional up-regulation^3^.

In this context, investigating the mechanisms associated with HSF1-induced HC acetylation and sat III transcription represents an important goal in understanding the biology of HC. Here, we report that HSF1 directs several Histone Acetyl Transferases to pericentric HC in stressed cells. We also bring evidence that Bromodomain-containing proteins BRD2, BRD3 and BRD4 are specifically and massively recruited to pericentric HC in heat-shocked cells. Accordingly, we provide new evidence that unlike hsp70 gene, JQ1 specifically inhibits sat III transcription by RNA polymerase II (RNAP II), pointing to the existence of important differences in the mechanisms of activation of two major targets of the heat shock response.

## Results

### Characterization of heat-induced acetylation at pericentric HC

The transcriptional activation of pericentric HC is probably one of the most striking events occurring in response to stress. Unlike hsp genes, transcriptional activation at pericentric HC is thought to occur within compact chromatin, and the molecular mechanisms involved are still poorly characterized. Interestingly, the presence of a large number of transcribed units at the 9q12 locus, resulting in the formation of nSBs about 1 µm diameter, allows a characterization of the actors involved in the activation of these regions by *in situ* approaches^23^.

**Figure 1:**
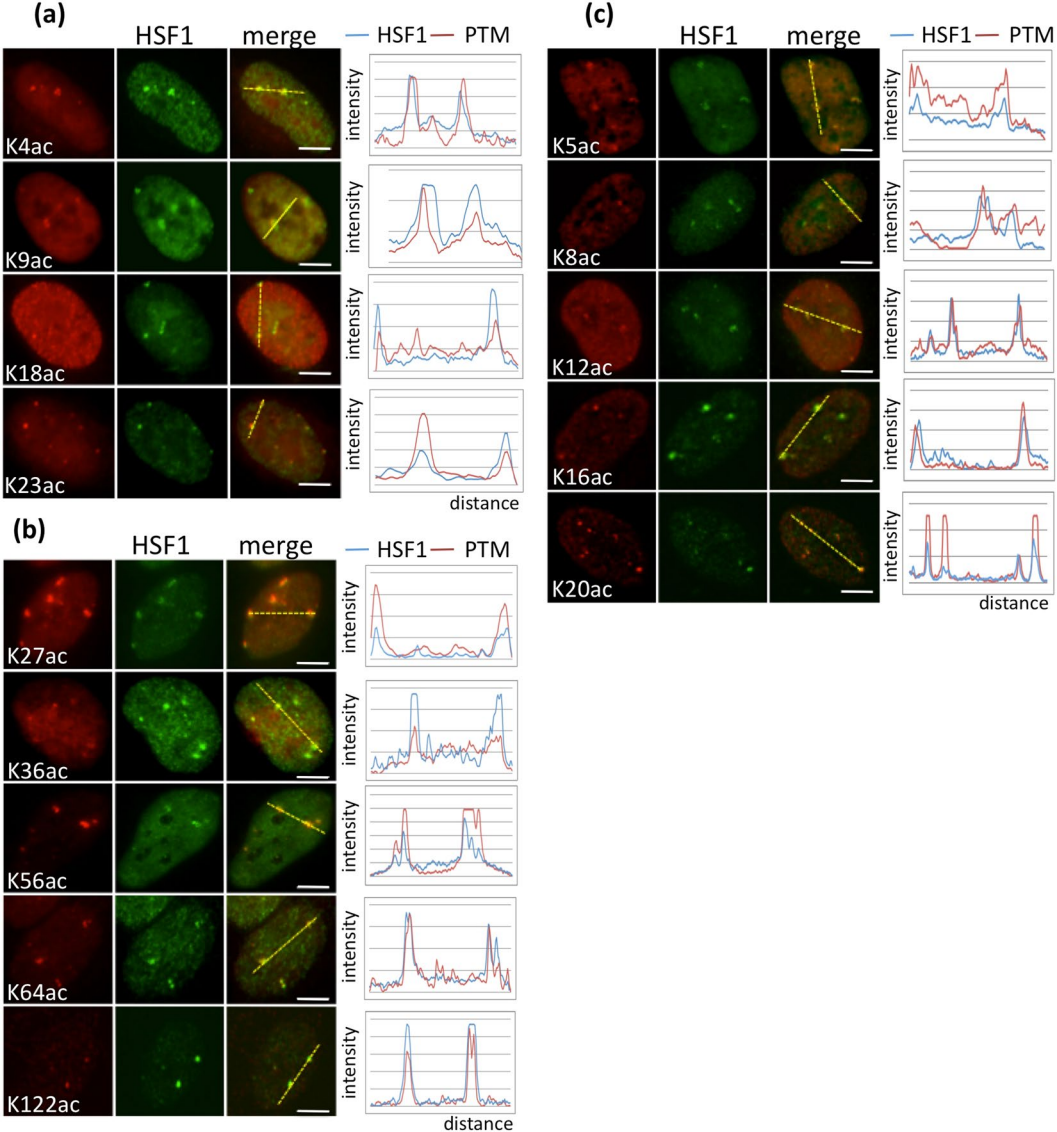
Status of active histones H3 and H4 marks at the 9q12 locus in HeLa stressed cells. (**a**,**b**) Active H3 and (**c**) H4 histone marks (red signals) and HSF1 (green signals) detected by immunofluorescence with fluorescence profiles (Scale bar = 5 µm). PTM = Post Translational Modification.

As a step toward a better characterization of chromatin acetylation at the 9q12 locus, analysis of specific histone marks was performed in heat-shocked cells. Unstressed cells display a homogenous distribution of acetylated chromatin (see Supplementary Fig. S1), while foci enriched in acetylated chromatin are detected in heat-shocked cells^2, 3^. As shown in Fig. 1a–c, acetylated lysine residues of histone H3 (K4, K9, K18, K23, K27, K36, K56, K64, K122) and H4 (K5, K8, K12, K16, K20) were enriched at nSBs in stressed cells (acetylated foci present in >90% of all nSBs) (HeLa cells are tetraploid for chromosome 9 so that more than two foci were detected in these cells). Stress-induced acetylation of histone residues at histone H3 and H4 at nSBs appears as a broad phenomenon since a broad range of acetylated residues are enriched at nSBs.

**Figure 2:**
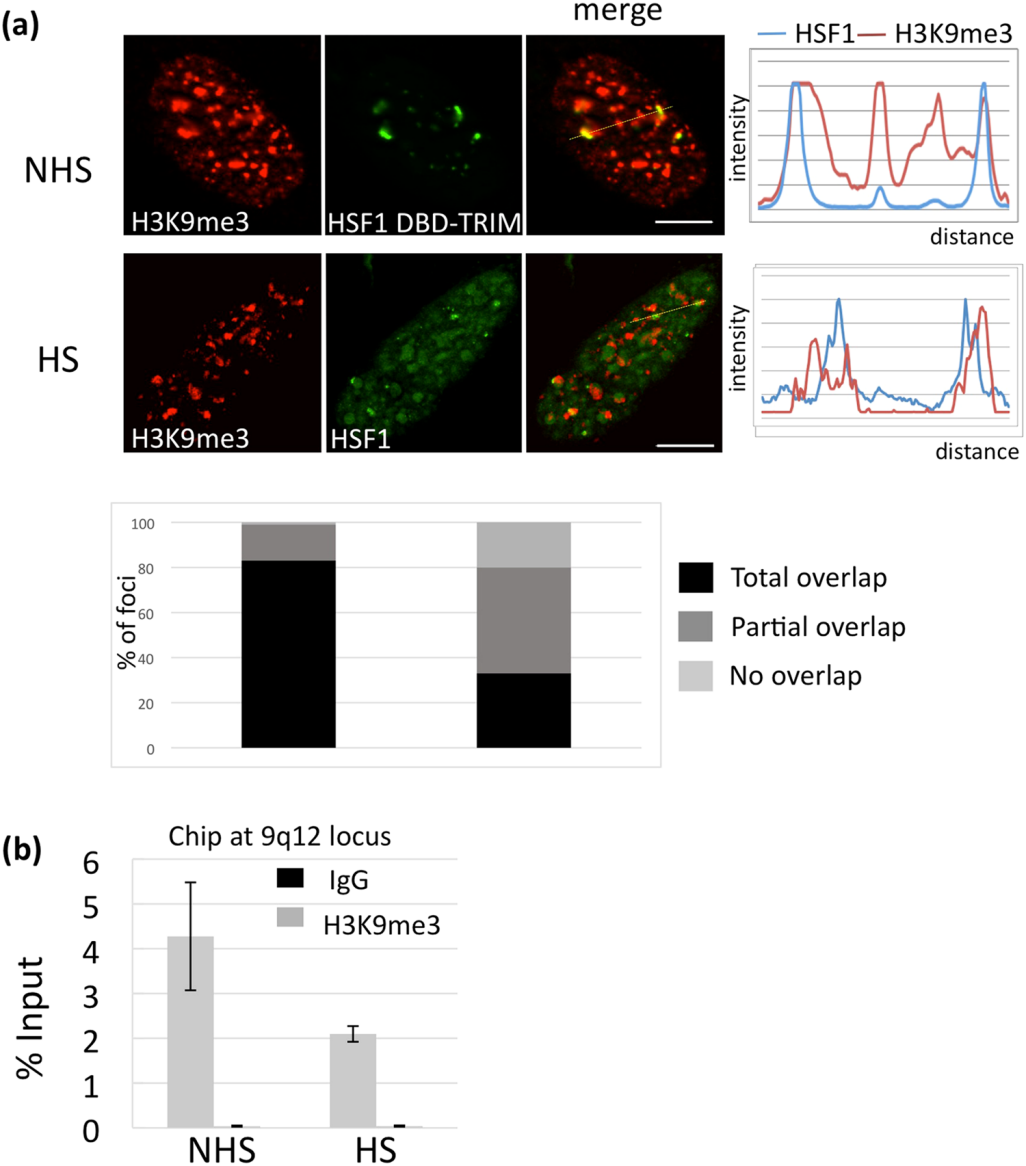
Loss of repressive H3K9me3 histone mark at the 9q12 locus in stressed HeLa cells. (**a**) H3K9me3 (red signals) was detected in unstressed cells with the DBD-TRIM-GFP HSF1 mutant and in stressed WT cells with endogenous HSF1 (green signals). In stressed cells, a higher percentage of foci displaying no overlap or a partial overlap of HSF1 (green signals) and H3K9me3 (red signals) fluorescence profiles is observed. Percentages were obtained from a total of 300 nSB profiles (Barr = 5 µm). (**b**) Evolution of H3K9me3 at the 9q12 locus determined by ChIP in HeLa unstressed (NHS) and stressed (HS) cells.

The status of the repressive histone mark H3K9me3 was also analyzed by IF (Fig. 2a) and ChIP (Fig. 2b) in unstressed and stressed cells. In unstressed cells, the 9q12 locus can be detected with a plasmid expressing a HSF1 mutant containing the trimerization (TRIM) and DNA binding (DBD) domains but devoid of the transactivating domain. This DBD-TRIM HSF1 mutant displays a constitutive binding capacity and behaves as a dominant negative factor^24^. This plasmid allows a direct identification of the 9q12 locus in unstressed cells, with no need to perform DNA FISH after acquisition of the IF image. As shown in Fig. 2a, H3K9me3 enrichment at the 9q12 locus is less pronounced in stressed cells. Of note, the absence of a global reduction of H3K9me3 was previously shown for the rest of the nucleus^25^. In both unstressed and stressed cells, the percentage of foci in which total, partial and no overlap between HSF1 and H3K9me3 fluorescence profiles was observed is given as a plot. Decrease of H3K9me3 labeling at the 9q12 locus was also confirmed by ChIP experiments (Fig. 2b). Our data thus suggest that stress-induced acetylation we observe at H3K9 (Fig. 1a) occurs at regions initially enriched in H3K9me3 and that acetylation probably occurs at the expense of H3K9 methylation.

### HSF1 triggers massive chromatin acetylation through interaction with Histone Acetyl Transferases

**Figure 3:**
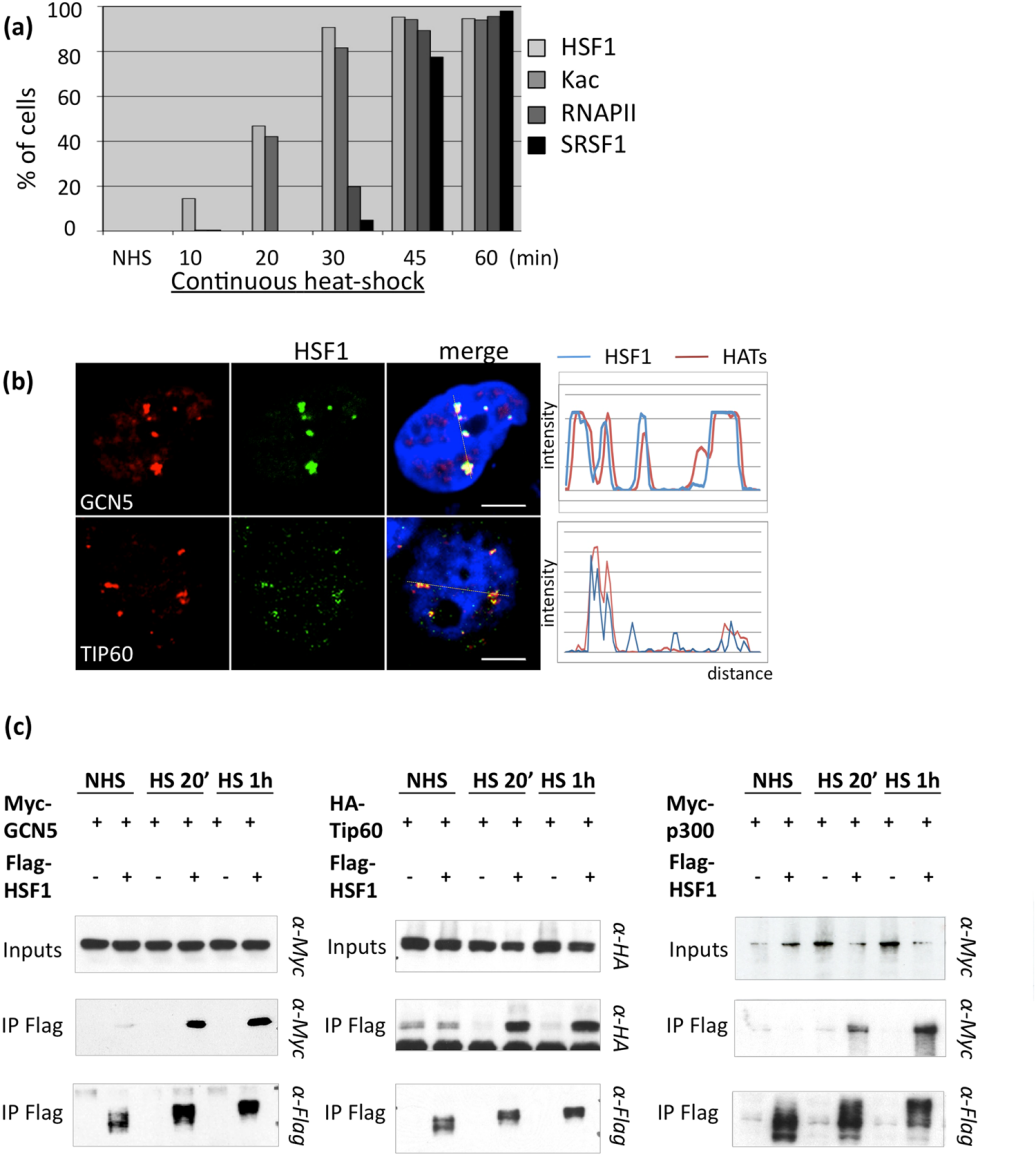
GCN5, TIP60 and p300 HATs are recruited to nSBs through their interaction with HSF1. (**a**) Percentage of cells displaying HSF1, acetylated histones, RNAP II and SRSF1 enrichment at nSBs over a kinetics of continuous heat shock exposure determined by immunofluorescence. Each percentage was calculated from a total of 300 cells. (**b**) Colocalization of endogenous HSF1 (green signals) and endogenous GCN5 or TIP60 (red signals) detected by immunofluorescence. Scale bar = 5 µm. (**c**) co-immunoprecipitation of Flag-HSF1 with HA-TIP60, Myc-GCN5 or Myc-p300. All three HATs were detected in Flag-HSF1 immunoprecipitates from unstressed and stressed Cos7 cells (Original blots are presented in Supplementary Figure S2).

To better characterize the kinetics of histone acetylation with regard to the kinetics of HSF1 binding and sat III transcription by RNAP II, cells were submitted to a continuous heat shock exposure of 10 to 60 min. The evolution of HSF1 accumulation, of histone acetylation and of elongating RNAP II was analyzed by immunofluorescence. SRSF1, a splicing factor binding to sat III RNA, was used as a marker of sat III RNA accumulation. Indeed, SF2/ASF enrichment is always detected at HSF1 foci enriched in Sat III RNA^26–28^, allowing an indirect detection of Sat III transcript by immunofluorescence. As shown in Fig. 3A, the presence of HSF1 and of acetylated foci was observed earlier in time at nSBs (10 and 20 min HS) than the detection of elongating RNAP II and SRSF1 (30 min). Moreover, the evolution of the percentage of nuclei displaying HSF1 and acetylated foci in response to a continuous heat exposure (80% of nuclei displaying both HSF1 and acetylated foci after 30 min of heat shock), suggests that both events are tightly correlated in time.

Three HATs were tested for their presence at nSBs in HeLa stressed cells. Besides p300/CBP already identified at nSBs^2^ and in HSF1 complexes in heat-shocked cells^22, 29^, two other HATs with pleiotropic roles, GCN5 and TIP60, were also detected at these bodies by IF (Fig. 3b). To further unravel the role played by active HSF1 in the recruitment of these three HATs, cells were further transfected with a plasmid expressing Flag-HSF1 and either Myc-GCN5, Myc-p300 or HA-TIP60. Immunoprecipitations of Flag-HSF1 were performed with extracts from Cos7 cells submitted or not to a 20 or 60 min heat shock. Immunoprecipitations of Flag-HSF1 were performed with extracts from Cos7 cells submitted or not to a 20 or 60 min heat shock. Indeed, Cos7 cells from green Monkeys do not display nSBs^30^, making these cells as an appropriate biological system to test the capacity of HSF1 to interact with the considered HATs, independently of nSBs formation.

As shown in Fig. 3c, specific HSF1/p300/GCN5 or/TIP60 complexes were detected only in extracts from cells submitted to a 20 or 60 min heat shock while not detected in control cells. The lowered HSF1 mobility observed in the 42 °C sample is due to stress-induced post-translational modifications of the protein. Co-IP experiments indeed indicate that, in these cells, the interaction between HSF1 and HATs we observe in stressed cells is independent of the presence of nSBs.

**Figure 4:**
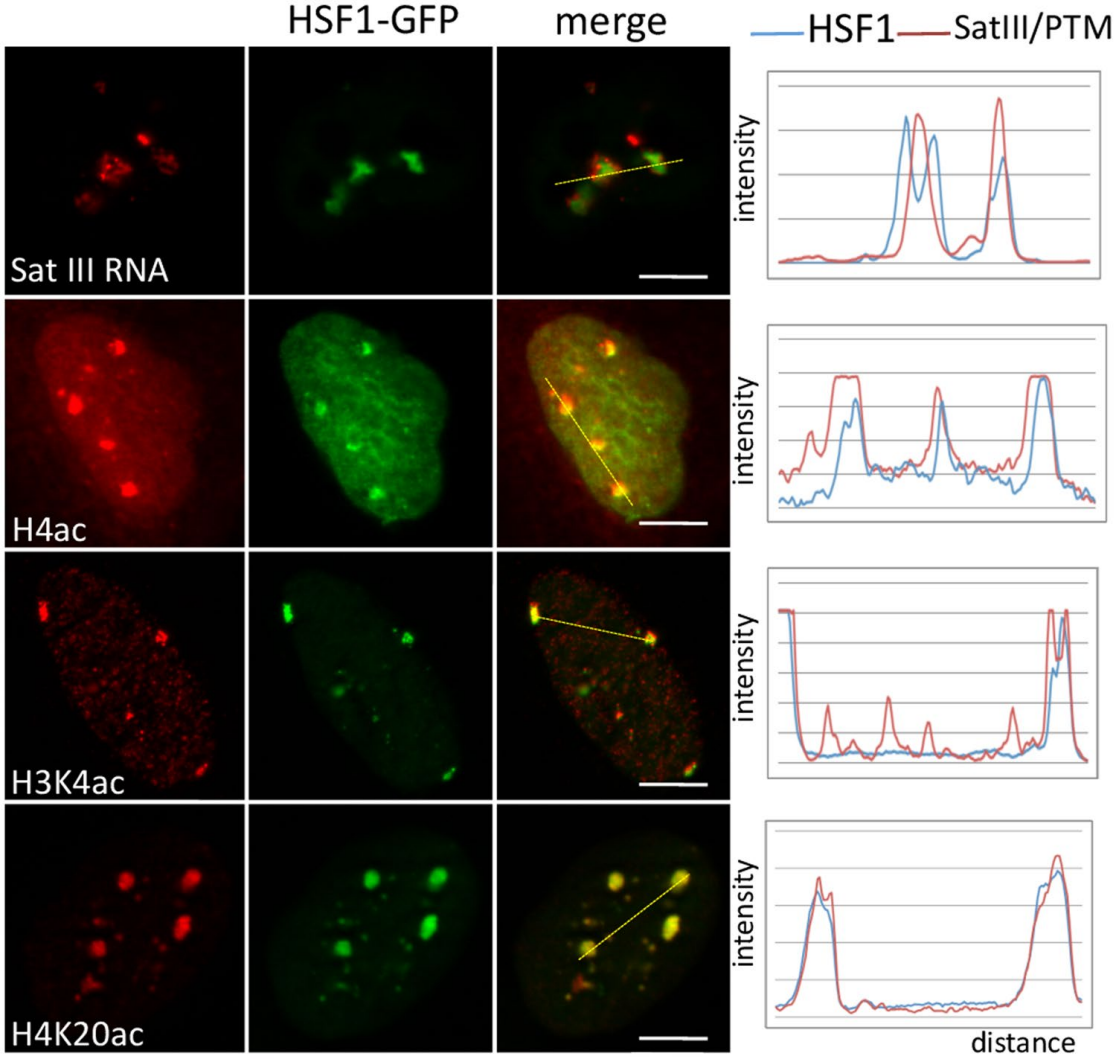
Overexpressed HSF1 directs chromatin acetylation at nSBs in unstressed HeLa cells. HSF1 foci were detected in cells transiently transfected with HSF1-GFP. HSF1-GFP foci correspond to regions of hyperacetylated chromatin and of sat III RNA accumulation, thus demonstrating that DNA-binding competent HSF1 directs chromatin acetylation at the 9q12 region in a stress-independent manner. Scale Bar = 5 µm.

HSF1 is negatively regulated by HSP70^31^ and, consequently, by a modification of the HSP70/HSF1 ratio in cells. Overexpressing HSF1 results in the constitutive HSF1 binding to its genomic targets, even in the absence of stress. To test whether HSF1 binding at the 9q12 locus was sufficient to promote an acetylation and transcription of the 9q12 locus in a heat-shock-independent manner, the transcriptional activation of the 9q12 locus, was tested in cells transfected with HSF1-GFP. As shown in Fig. 4, HSF1-GFP foci were present in a fraction of these cells. These foci were enriched in acetylated histones and in sat III transcripts, suggesting that overexpressed HSF1 is able to recruit HATs and to drive acetylation and transcription, when overexpressed, in a heat-independent manner. However, it is interesting to note that while acetylated foci, in HSF1 overexpressing cells, are present at the 9q12 locus, only interaction between GCN5 and HSF1 was detected in HSF1 immunoprecipitates from stressed cells. The reason could be that only a low percentage of transfected cells express HSF1 at a level sufficient to trigger HSF1 binding in the absence of stress and/or that the interaction between HATs and HSF1 is weaker in unstressed cells.

### BET family members play an essential role in pericentric HC activation upon stress

**Figure 5:**
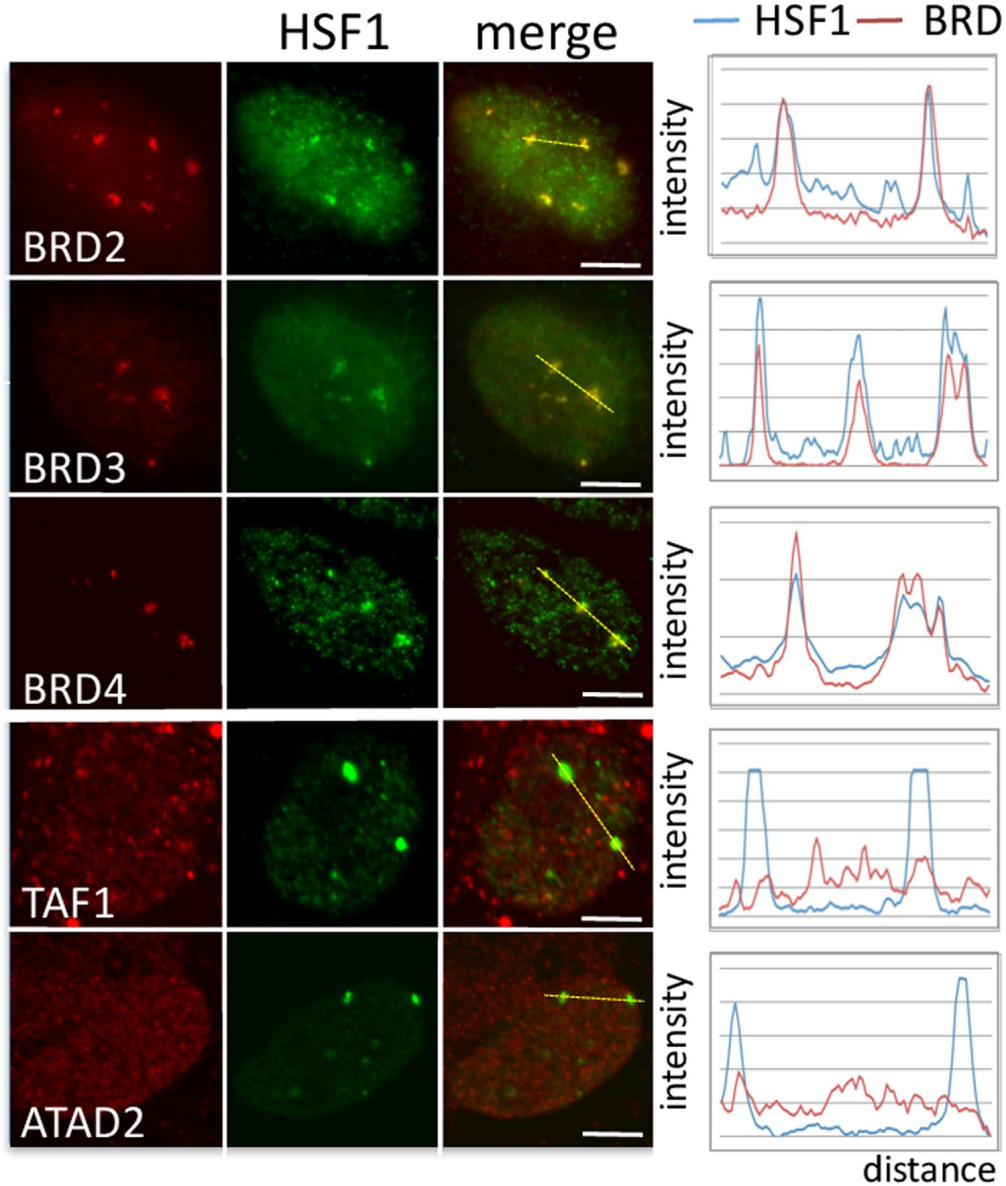
Specific Bromodomain-containing proteins are recruited to the 9q12 locus in stressed HeLa cells. Bromodomain containing proteins (green signals) are co-detected with HSF1 (red signals) by immunofluorescence on stressed cells. Endogenous BRD2, BRD3 and BRD4 are enriched at nSBs together with endogenous HSF1 (green signals) while TAF_250_ and ATAD2 are not. Absence of TAF1 and ATAD2 enrichment at the 9q12 locus was confirmed by comparative analysis of TAF1 or ATAD2 and HSF1 fluorescence profiles. Scale bar = 5 µm.

Bromodomain-containing proteins represent important actors in transcription initiation, and it has been shown that certain bromodomains target specific histone acetylation^17^. Because of the wide range of acetylated lysines detected at nSBs, we wondered if BRD proteins could also be recruited on pericentric HC upon HS. The distribution of the BET family members BRD2, 3, 4, and of two other BRD containing proteins ATAD2 and TAF1/TAF250 was analyzed in stressed cells and their recruitment to nSBs examined. Endogenous BRD2, BRD3, BRD4 were clearly enriched at nSBs in stressed cells (colocalization with HSF1 foci >90%) while no recruitment of the TFIID subunit TAF250, and of the chromatin modulator ATAD2^32^, was observed, revealing the presence of certain, but not all, bromodomain proteins to nSBs (Fig. 5 and see Supplementary Fig. S3).

**Figure 6:**
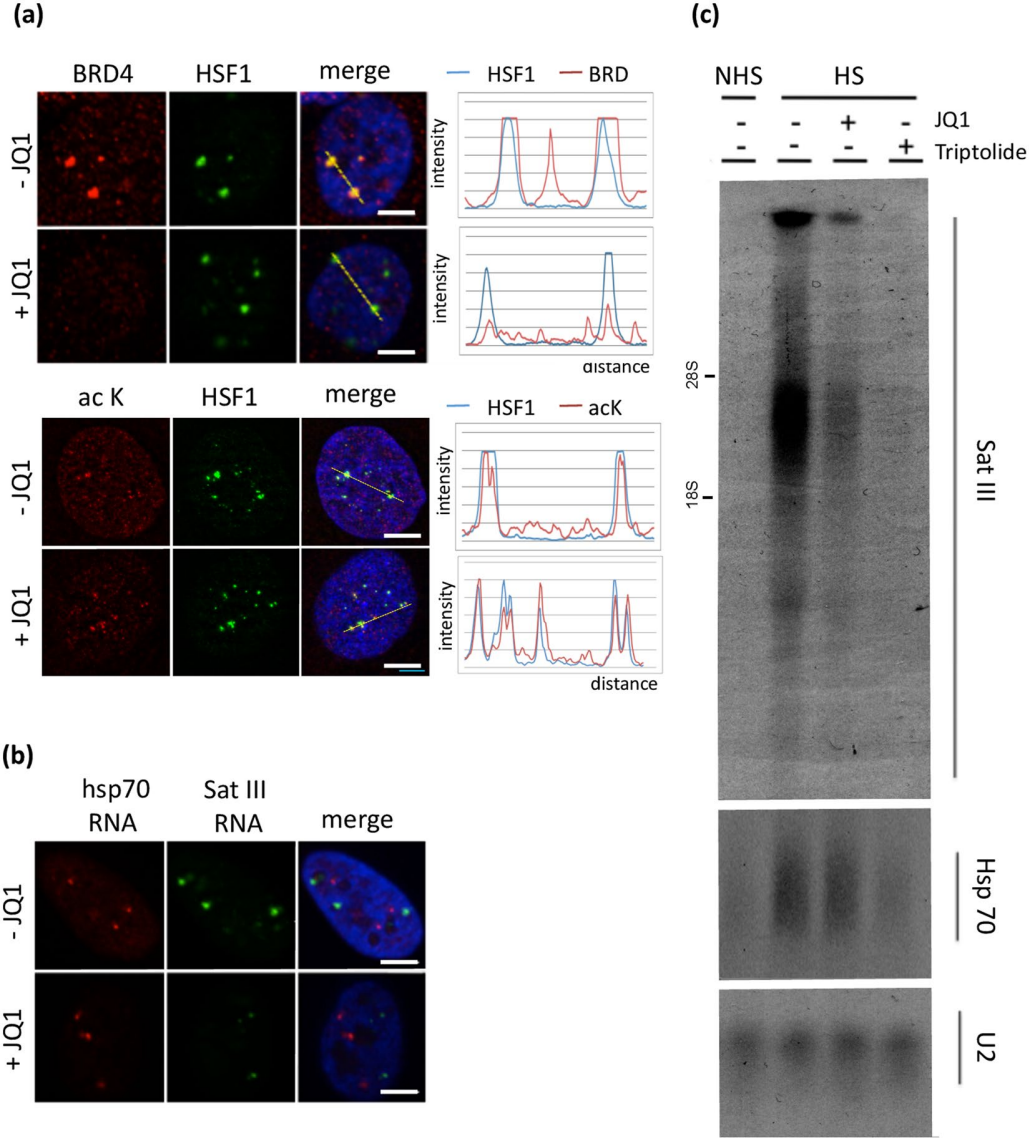
Inhibition of Brd proteins binding to acetylated chromatin by JQ1 impairs the transcriptional activation of sat III sequences by RNAP II in stressed cells. (**a**) HSF1 (green signals) was either co-detected with BRD4 by immunofluorescence (red signals) (upper image) or detected with acetylated Lysine (lower image) (**b**) hsp70 (red signals) and sat III (green signals) RNA were detected with RNA FISH (same exposure time were used in both conditions). (**c**) Impact of JQ1 on hsp70 and sat III RNA accumulation by Northern blot on RNA fractions prepared from unstressed (NHS) and stressed (HS) cells treated or not with JQ1 (original blots are displayed in Supplementary Figure S5).

**Figure 7:**
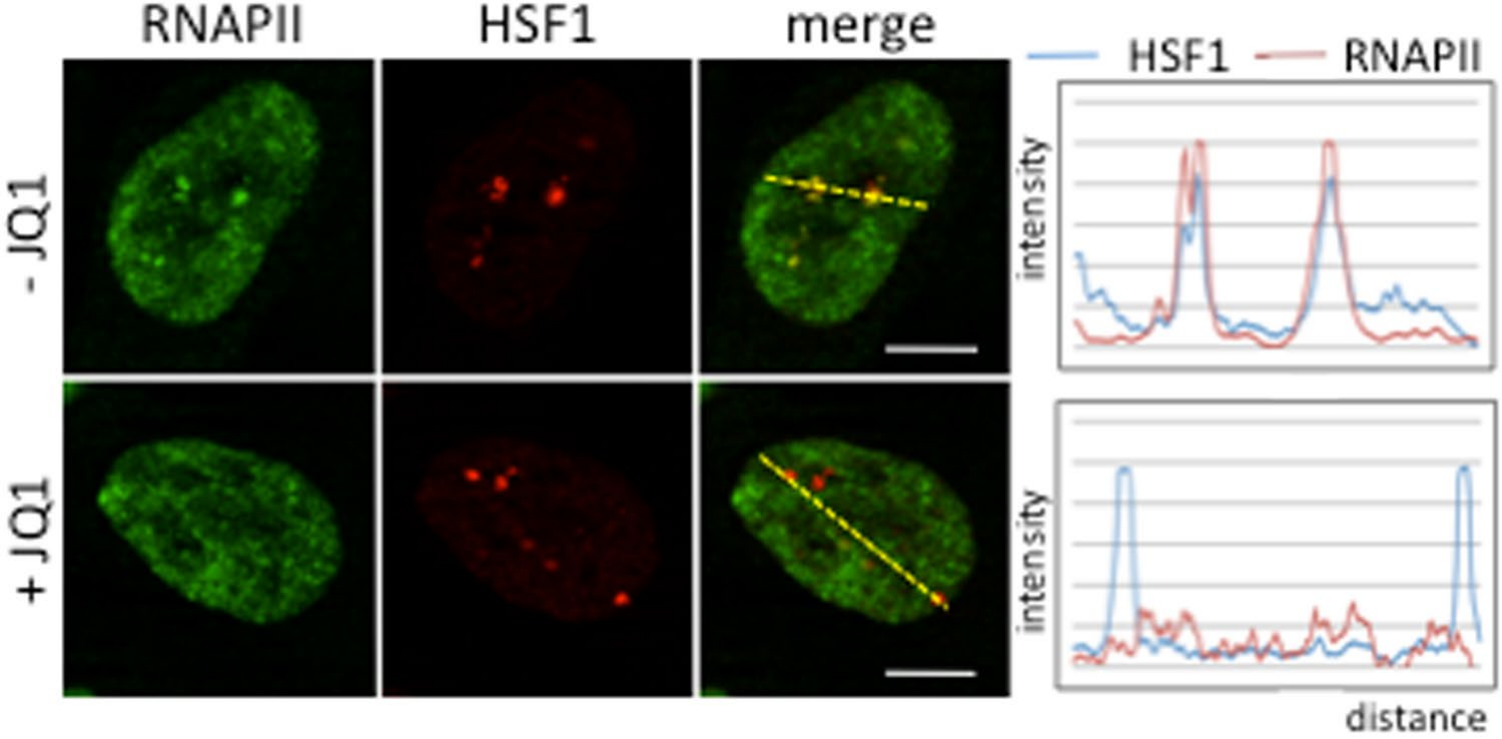
Detection of endogenous HSF1 and RNAP II by immunofluorescence in stressed cells treated or not with JQ1. Colocalization between RNAP II (green signals) and HSF1 (red signals) is >90% in the absence of JQ1 and <10% in cells treated with JQ1. Scale bar = 5 µm.

To determine the functional implication of BET proteins in HSF1-mediated sat III transcription, the impact of JQ1, a specific inhibitor of BET binding on acetylated histones, was tested on HSF1 foci formation, on chromatin acetylation at the 9q12 locus, and on hsp70 and sat III transcription. As expected, the recruitment of BRD4 (Fig. 6a), BRD2 and BRD3 proteins (see Supplementary Fig. S3) was lost on nSBs in the presence of JQ1, thus validating the efficacy of the JQ1 treatment in stressed cells. In contrast, HSF1 targeting to nSBs and the formation of acetylated foci was not affected by JQ1 (Fig. 6a). This clearly demonstrated that BET proteins do not play a major role, neither in HSF1 recruitment to and stabilization at the 9q12 locus in stressed cells, nor in HSF1-mediated HC acetylation. In contrast, a significant decrease in the amount of sat III RNA was observed in JQ1 treated cells, both by RNA FISH (Fig. 6b) and Northern blot experiments (Fig. 6c). This shows that, as expected, BET proteins act on HC after HSF1 binding and subsequent chromatin acetylation. This also shows that impairing the recruitment of BET proteins to acetylated chromatin by JQ1 affects sat III transcription. Surprisingly, while Triptolide, a potent inhibitor of RNA Pol II initiation both impaired the stress-induced accumulation of HSP70 and Sat III transcripts, no impact of JQ1 on the stress-induced accumulation of HSP70 transcripts was observed (Fig. 6b and c), therefore identifying BET proteins as essential actors in sat III (and not hsp70) transcriptional activation, and JQ1 as a potent new decoupling agent of HSP70 and sat III transcription in heat-shocked cells. While no impact of JQ1 on histone acetylation at the 9q12 locus was observed, no accumulation of RNAP II (either phospho or not phosphorylated) at this locus was detected in JQ1 treated cells, revealing a new important role of BRD proteins in the recruitment of RNAP II at pericentric HC of stressed cells. (Fig. 7).

## Discussion

One of the most dramatic examples of transcriptional activation of heterochromatic regions is the one occurring in response to cell stress. The heat shock response thus offers a unique model to explore the epigenetic mechanisms underlying pericentric HC activation that may also be involved in the different contexts where such activation occurs, including early development^33^, senescence^34^ or cancer^35^.

The capacity of HSF1 to rapidly and efficiently activate the transcription of HC very likely relies on its capability, shown here, to interact with HATs, and to drive them to pericentric HC. Coimmunoprecipitation of p300/CBP with HSF1 has already been reported^22, 29^ and the enrichment of CBP at nSBs^2^ also described. We extend here these findings to two other essential transcriptional co-activators belonging to two additional HAT families: GCN5, and TIP60 and have also identified the domains possibly involved in their interaction with HSF1 (see Supplementary Fig. S4). As for p300, we have no evidence so far that TIP60 and GCN5 interaction with HSF1 is direct. The high number of acetylated histone marks found at nSBs however strengthens the idea that chromatin acetylation at nSBs results from the active targeting of HATs through a HSF1-driven process. It also reinforces the possibility that a massive recruitment of HATs at nSBs may deplete them from the rest of the nucleus and participate to the global down regulation of gene expression occuring in heat-shocked cells^23^. The transcriptional activation of sat III by HSF1 could thus represent a powerful way to trigger a rapid remodeling of gene expression at a genome-wide scale in heat-shocked cells. Our observation also reveals that the enrichment of H3K9me3 at pericentric HC is not a barrier to HSF1 binding at these regions and to the HSF1-dependent transactivation of these loci. In the yeast *S. cerevisiae*, SIR-generated HC, although devoid of H3K9me3 histone marks, is permissive to the binding of HSF and components of the preinitiation complex (PIC), TBP and RNAPII, in a model where a heat shock gene was flanked by mating-type silencers^36^. We now broaden these observations to an endogenous locus in human cells, and confirm the capacity of HSF1 to promote a rapid deposition of active epigenetic marks at regions enriched in H3K9me3, thus reinforcing the classification of HSF1 in the category of pioneer transcription factors, able to access target element in nucleosomal DNA when other factors cannot^10, 37^.

Acetylation of all four H2A, H2B, H3 and H4 histones at nSBs has already been reported, more specifically at H4K8 and K16^2, 3^ and is consistent with the active transcriptional state of HC upon HS. We show that acetylation targets lysine residues at the N terminal histone tails but also, in the case of H3K56, H3K64, H3K122, at histone fold motifs. Among the different acetylation marks we detect at nSBs, three of them have been related to heterochromatin maintenance. H3K4ac has been proposed to regulate heterochromatin formation, and more specifically the switch between active and inactive HC formation at S phase in the yeast *S*. *pombe*, allowing the binding of Chp2 and Swi6, the yeast homologs of HP1, to H3K9me2/3^38^. A very recent work now also points to the contrasting role of two other acetylation marks in mammals. Indeed, H3K18 deacetylation at pericentric HC has been found to stabilize pericentric HC and to prevent mitotic errors and cellular senescence^39^ while, conversely, a new study points to the importance of TIP60-dependent H3K12 acetylation, through BRD2 interaction, to stabilize pericentric HC in Suv39H−/− mouse cells^40^.

The contrasting roles of histone acetylation in these different biological contexts, might reflect the dual roles of pericentric HC activation. Indeed, in stressed cells, pericentric HC transcriptional activation and sat III RNA accumulation may serve, through the transient recruitment of transcription factors and of RNA maturation factors at nSBs, as an efficient way to trigger global and rapid changes in gene expression. Conversely, transient accumulation of pericentric RNA also participates, as in the yeast *S*. *pombe* ^41^ and mammals^33, 42^ in HC maintenance and establishment through the recruitment of proteins, such as HP1 (Heterochromatin Protein 1)^42, 43^. Finally, based on the recent finding that deacetylation of H3K18 at pericentric HC seems to prevent mitotic errors and cellular senescence^39^, histone acetylation at pericentric HC may promote a rapid activation of cell cycle checkpoints and be used as a sensor of stress.

Our observation that HSF1 overexpression is sufficient to trigger chromatin acetylation and sat III accumulation at nSBs suggests that active HSF1 is able to drive HATs to nSBs, even in the absence of stress, with possible impact in tumors where HSF1 overexpression and nuclear localization is associated with reduced cell survival^44^.

**Figure 8:**
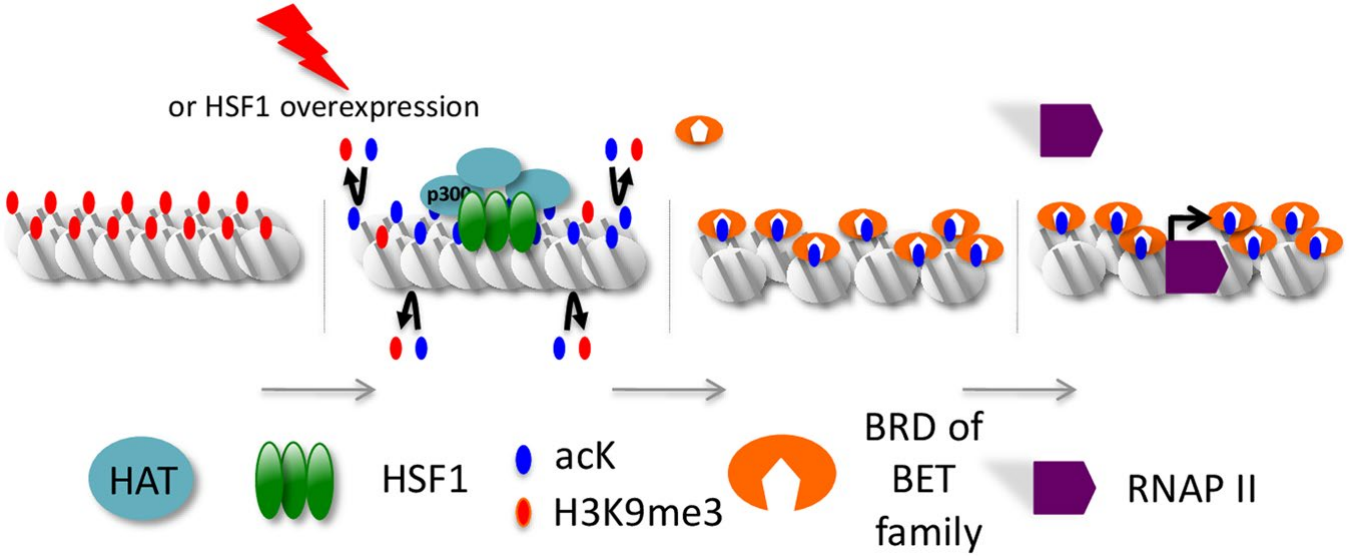
General scheme of sat III transcription at nSB. Heat shock allows a massive recruitment of several HATs to sat III sequences through their direct or indirect interaction with HSF1. Acetylated lysine residues of histones H3 and H4 then serve as docking sites for Bromodomain proteins of the BET family, triggering chromatin remodeling and RNAP II recruitment.

Acetylated residues within the N terminal tails and histone fold motifs neutralize positive charges on histones and/or serve as platforms for the recruitment of essential transcription factors. Our data now point to the important role of acetylation in the selective recruitment of Bromodomain proteins BRD2, BRD3, and BRD4 on nSBs, with a role in sat III accumulation. Our finding that JQ1 selectively impairs the accumulation of sat III and not that of hsp70 transcripts reveals major differences in the mechanisms underlying sat III and hsp70 gene transcription. Differences of chromatin compaction between hsp70 gene and sat III sequences could set the basis for the differences of the JQ1 impact we observe. The BRD2 protein has been found to display an intrinsic histone chaperone activity, rendering nucleosomes marked by acetylation, permissive to the progression of elongating RNAPII^16^. Likewise, BRD4 activates transcriptional elongation through different ways. One, specifically found at enhancers, relies on the acetyl binding property of the BRD4 bromodomain and involves the histone chaperone activity of BET proteins^45^. A second way, not impaired by JQ1, would rely on the capacity of BRD4 to recruit the P-TEFb complex allowing RNAP II phosphorylation and release from pausing at transcription start sites^46^. The new observation we make that RNAP II accumulation at the 9q12 locus is impaired in JQ1 treated cells, reveals a new specific role for Brd proteins in RNAP II recruitment at pericentric HC in stressed cells and indicates that the histone chaperone function of BET proteins is preferentially required for HC transcription (Fig. 8). Indeed although pTEFb is known to play a role in the elongation of RNAP II at hsp70 gene^47–49^, the role of BET proteins in pTEFb recruitment on Hsp70 promoter, if any, is not impaired by JQ1.

Additional and non-exclusive functions of BET proteins could also be envisioned. In *S. pombe*, Bdf2, the yeast homolog of BRD4 has been found to create a barrier between the bunch of active chromatin and adjacent regions. One cannot exclude that the recruitment of BET proteins to heterochromatic regions could favor the isolation of the active locus from its adjacent environment^50^. Moreover, while our paper was under reviewing, a study reported that Brd4 recruitment to pericentric HC could protect genes from splicing inhibition upon heat shock. This study therefore also identifies pericentric HC as a new specific target of BRD proteins in heat-shocked cells^51^. The possibility to uncouple sat III and hsp70 gene transcription should help us to unravel the specific role of pericentric activation and sat III transcripts in heat-shocked cells and in the various different physiopathological contexts, such as cancer^23, 52, 53^, for which an accumulation of sat III RNA has been detected.

## Material and Methods

### Cell culture, heat shock and drug treatments

#### Cell lines

Immunofluorescence and ChIP analysis were performed in HeLa cells, from cervix adenocarcinoma (American Type Culture Collection, VA, USA). Co-immunoprecipitations were performed in fibroblast-like Monkey Cos7 cells.

Heat-shock experiments were performed by immersion of the culture flasks in a warm water bath. Unless otherwise stated, heat-shock was performed for 1 h at 43 °C.

JQ1 treatment^19^ was achieved by treating the cells at a concentration of 500 nM, 5 min before heat shock.

Transient transfections were performed using lipofectamine®2000 solution (ThermoFisher Scientific).

Transcription inhibition was performed with adding Triptolide, an inhibitor of RNAP II initiation^54^, at a final concentration of 5 mM in the culture medium 5 min before stress. Triptolide was then maintained during the heat-shock treatment.

### Plasmids

Expression plasmids for human CBP-Ha, Ha-Gcn5 and p300 were provided by A. Harel-Bellan (Institut A. Lwoff, Villejuif, France), and H. Stunnenberg (Radboud University Nijmegen, The Nederland), respectively. Human pcDNA Ha‐tagged TIP60 expression plasmids are described in^55, 56^. pH2.3 clone corresponding to human hsp70 sequence^57^ was provided by R.I. Morimoto (Northwestern University, Evanston, IL, USA). The plasmids expressing the mouse HSF1-GFP, DBD-TRIM-GFP and FLAG-HSF1 were developed in our laboratory^24, 58^. Detection of hsp70 and sat III RNA was performed with plasmid pH2.3 and pHuR98^57, 59^.

### Immunofluorescence, RNA FISH, DNA FISH, and microscopy

Immunofluorescence was performed on formaldehyde-fixed cells as previously described^58, 60^, with the following antibodies: rabbit anti-HSF1 (Enzo, SPA-950) (1:100), mouse anti-HSF1 (Santa Cruz sc 11757, 1:200), rabbit anti-H3K4ac (Millipore, 07–539, 1:100), rabbit anti-H3K9ac Abcam ab39917 (1:200), rabbit anti-H3K18ac Abcam ab1191 (1:50), rabbit anti-H3K27ac active motif 39133 (1:800), rabbit anti-H3K56ac Epitomics 2134-1 (1:250), rabbit anti-H3K64ac Active Motif 39546 (1:1000), rabbit anti-H3K122ac R. Schneider (1:100), rabbit anti-H4 K5ac Epitomics 1808-1 (1:500), rabbit anti-H4K8ac Epitomics 1796-1 (1:800), rabbit anti-H4K12ac (Millipore, 07–595, 1:500), rabbit anti-H4K16ac Millipore 07–329 (1:200), rabbit anti-H4K20ac Abcam ab19855 (1:200), anti-acetyl lysin Millipore, 06–933 (1:200), anti-Gcn5 Millipore 07–038 (1:50), anti-Tip60 Santa Cruz sc-20698 (1:100), anti-Brd2 (Abcam, 1:400), anti-Brd4 (Abcam, Ab75898) (1:300), and mouse anti-ATAD2 (Sigma, SAB1400512) (1:200). Anti-RNAP II Ser 2 (1:400) was obtained, from M. Vigneron (IGBMC, Strasbourg, France) (1:400). Anti-TAF1 was kindly provided by L. Tora (IGBMC, Strasbourg, France). Anti-Brd3 (1:100) was made by immunizing a rabbit with the peptides QLKKGGKQASASYDSC and CQAAKSKEELAQEK, followed by immuno-purification of the serum on the immobilized peptides.

Secondary anti-rat or -rabbit antibodies conjugated to either Dylight 549 (KPL) or Alexa 488 (Molecular Probes) were used. DNA was counterstained with DAPI (250 ng/mL). RNA FISH alone and in combination with immunofluorescence or DNA FISH was performed as described previously^60^. Cells were analyzed using an apotome microscopy system (Zeiss, Jena, Germany) or a confocal laser scanning microscope (CLSM) (LSM 510 META NLO, Zeiss) using a Plan-Apochromat 63x Oil objective (NA = 1.4). Confocal excitation wavelengths were 488 nm (FITC), 543 nm (Cy3), and 730 nm (DAPI- biphotonic excitation). The pinhole diameter was set to 1 Airy Unit for the confocal channels and fully opened for 2P excitation.

### RNA extraction and Northern blot

Total RNAs were extracted with Tri-reagent® (Sigma-Aldrich) following the manufacturer’s instructions. For Northern blot analysis, 10 µg of RNA were loaded on a 1% agarose denaturing gel. Before transfer, the gel was treated with 75 mM NaOH for 10 min, and washed in 0.5 M Tris/1.5 M NaCl, pH 7.0. The RNAs were then transferred onto Hybond N membrane (Amersham Biosciences) and hybridized with pH2.3^57^ or pHuR98^59^ probe labeled with- α32 P dCTP.

### Immunoprecipitation

Cos cells were plated in 10 cm dishes and transfected with 3 μg of a pcDNA-FLAG plasmid expressing a Human HSF1 (FLAG in Cter) or an empty vector as negative control, and a plasmid expressing either HA-p300 (5 µg), HA-GCN5 (4 µg) or HA-TIP60 (4 µg). Cells were lysed 24 h after transfection by incubation for 1 h on ice in LSDB500 (500 mM KCl, 20% glycerol, 3 mM MgCl2 and 50 mM Hepes pH 7.9) containing 0.2% NP‐40, 1 mM DTT, 200 μg/ml DNaseI, 100 μg/ml RNaseA, 50 ng/ml TSA and protease inhibitors cocktail (complete mini EDTA‐free; Roche). After centrifugation, the lysate was incubated with 1 μg of anti‐FLAG antibody (Sigma) for 1 h on ice. Protein G‐Sepharose beads (Amersham) were then added and incubated at 4 °C on a rotoshake for 1 h. After three washes with LSDB250 (same as the lysis buffer with 250 mM KCl), complexes were recovered by adding Laemmli sample buffer and analyzed by Western blots. Western blot was performed with a rat anti-HA (3F10) antibody (Roche).

### Chromatin immunoprecipitation (ChIP)

Cells were cross-linked with 1% formaldehyde at room temperature for 10 min before the addition of 125 mM glycine (Sigma) at room temperature for 5 min. After washing, cell were resuspended into cytosol lysis buffer (10 mM Hepes pH 6.5, 0.25% Triton-X100, 0.5 mM EGTA, 10 mM EDTA) at 4 °C for 5 min. Nuclei were resuspended in nuclei lysis buffer (50 mM Tris pH 8, 120 mM NaCl, 5 mM EDTA and 0.5% NP- 40) and then sonicated at 4 °C for 19 min with a BioRuptor sonicator (Diagenode) to obtain fragments between 200 and 800 base pairs. Samples were incubated overnight with the anti-H3 (Abcam), anti-H3K9Me3 (Abcam), anti-IgG (Diagenode) antibodies and immunoprecipitated using the OneDay ChIP kit (Diagenode), following the manufacturer instructions. Immunoprecipitated DNA was analyzed by qPCR, using primers. IP-PHUR1 (Fwd): AATCAACCCGAGTGCAATCGAATGGAATCG and IP-PHUR2 (Rev) TCCATTCCATTCCTGTACTCGG)^52^.

Standard deviations were calculated from 3 independent experiments.

## Electronic supplementary material

Supplementary Figures
