# Supplementary material for: Bromodomain factors of BET family are new essential actors of pericentric heterochromatin transcriptional activation in response to heat shock
Source: Sci Rep. 2017 Jul 14;7:5418. doi: 10.1038/s41598-017-05343-8 (PMC5511177; doi:10.1038/s41598-017-05343-8)
Supplement: Supplementary file 1 — Supplementary Figures [file 41598_2017_5343_MOESM1_ESM.pdf]

Bromodomain factors of BET family are new essential actors of pericentric heterochromatin transcriptional activation in response to heat shock

Authors : Edwige Col[1,6], Neda Hoghoughi[1,6], Solenne Dufour[1], Jessica Penin[1], Sivan Koskas[1], Virginie Faure[1], Maria Ouzounova[2], Hector Hernandez-Vargash[2], Nicolas Reynoird[1], Sylvain Daujat[4], Eric Folco[1], Marc Vigneron[3], Robert Schneider[4, 5], André Verdel[1], Saadi Khochbin[1], Zdenko Herceg[2], Cécile Caron[1], Claire Vourc'h[1]*

([1]) Université Grenoble Alpes, CNRS UMR 5309, INSERM U1209, Institute for Advanced Biosciences (IAB), Site Santé - Allée des Alpes 38700 La Tronche, France
([2]) International Agency for Research on Cancer (IARC), 69008 Lyon, France
([3]) UMR 7242, Ecole Supérieure de Biotechnologie de Strasbourg (ESBS), 300 boulevard Sebastien Brant, CS 10413,
67412 ILLKIRCH, France
([4]) Institute of Genetics and Molecular and Cellular Biology (IGBMC), Strasbourg, France
([5]) Institute of Functional Epigenetics, Helmholtz Zentrum Muenchen, Ingolstaedter Landstr 1, 85754 Neuherberg.Germany
([6]) These authors contributed equally to this work.
(*) Correspondence to: claire.vourch@univ-grenoble-alpes.fr

Supplementary Fig.S1

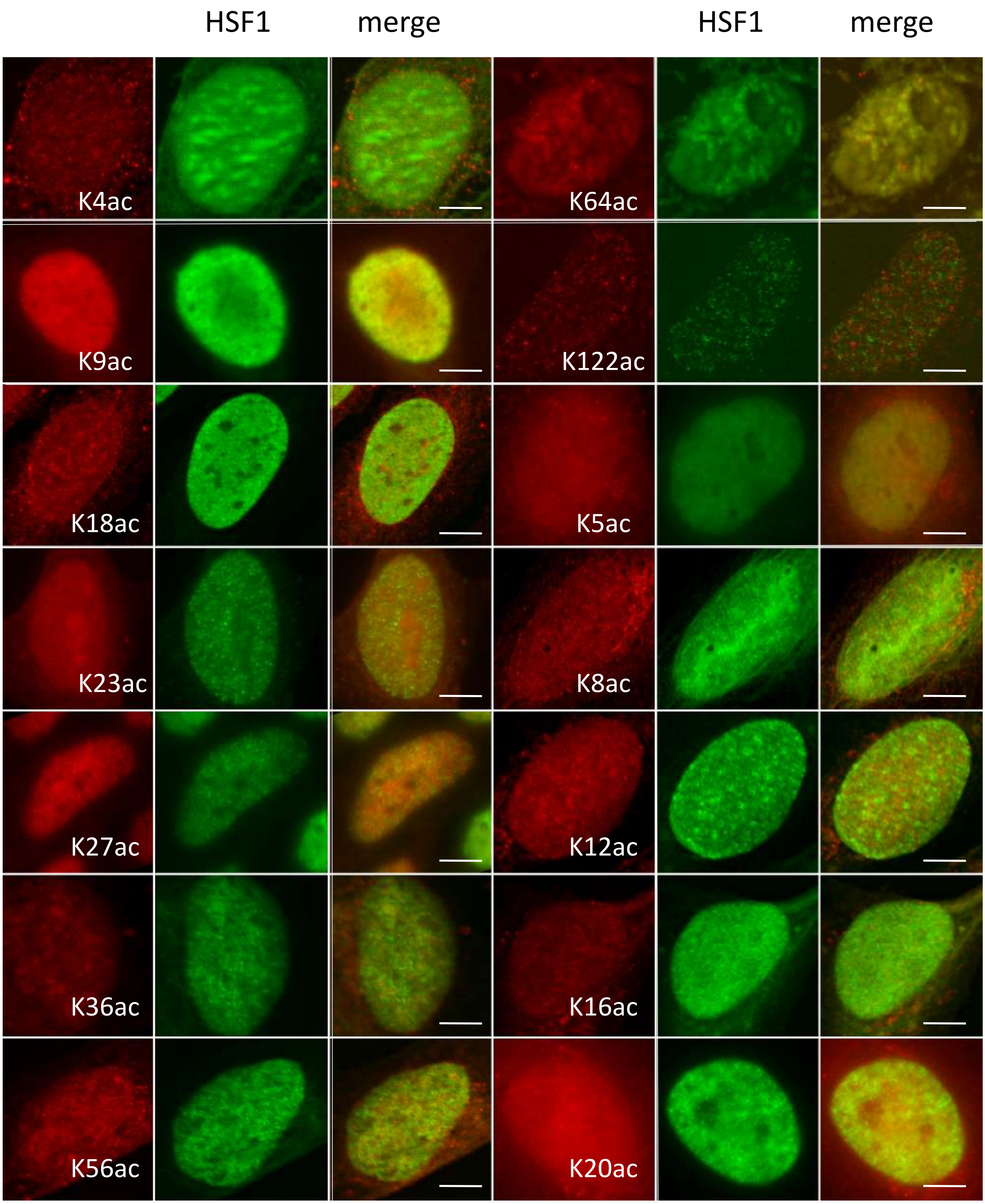

Supplementary Figure S1

Detection of specific acetylated H3 and H4 residues in unstressed HeLa cells. Acetylaled residues (red signal) detected with endogenous HSF1 (green signal). (All antibodies are listed in the Material and methods section) (Bar = 5 µm)

Supplementary Figure S2

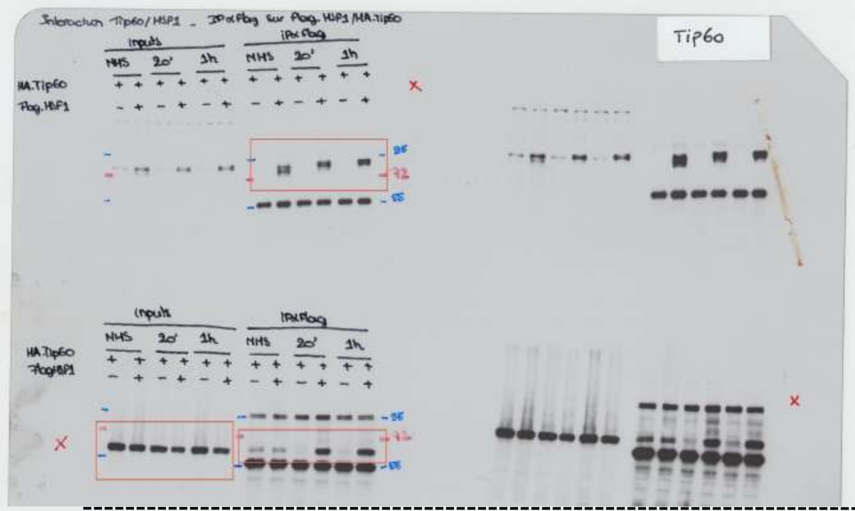

Tip60

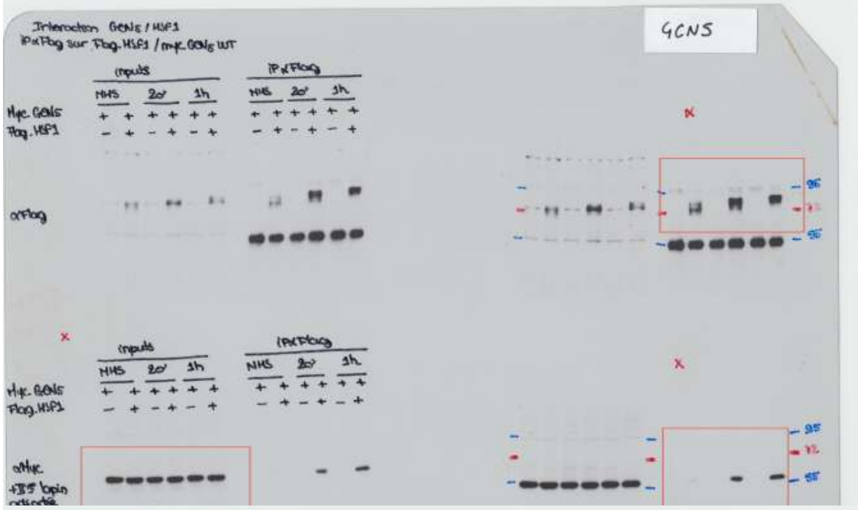

Gcn5

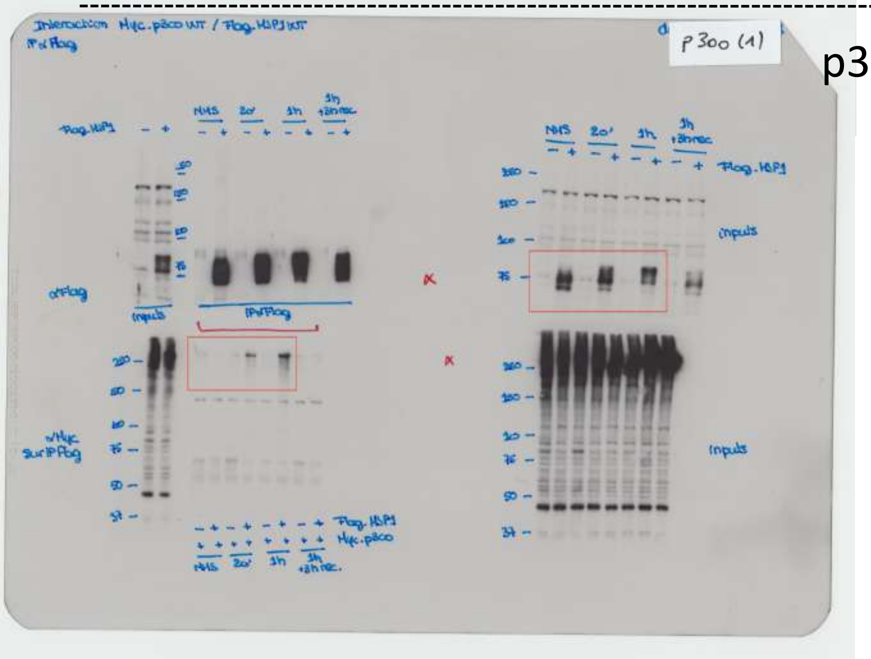

p300

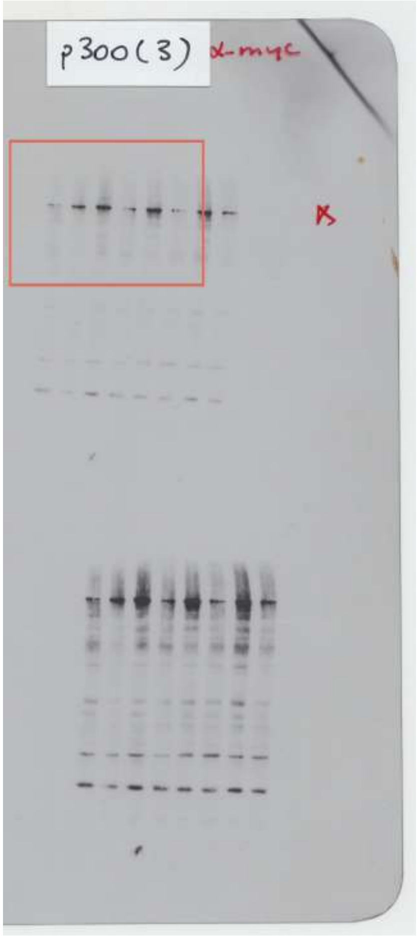

Supplementary Figure S2

Original autoradiograpy images of the western blots presented in Figure 3. The areas presented in Figure 3 are outlined with red frames.

Supplementary Fig.S3

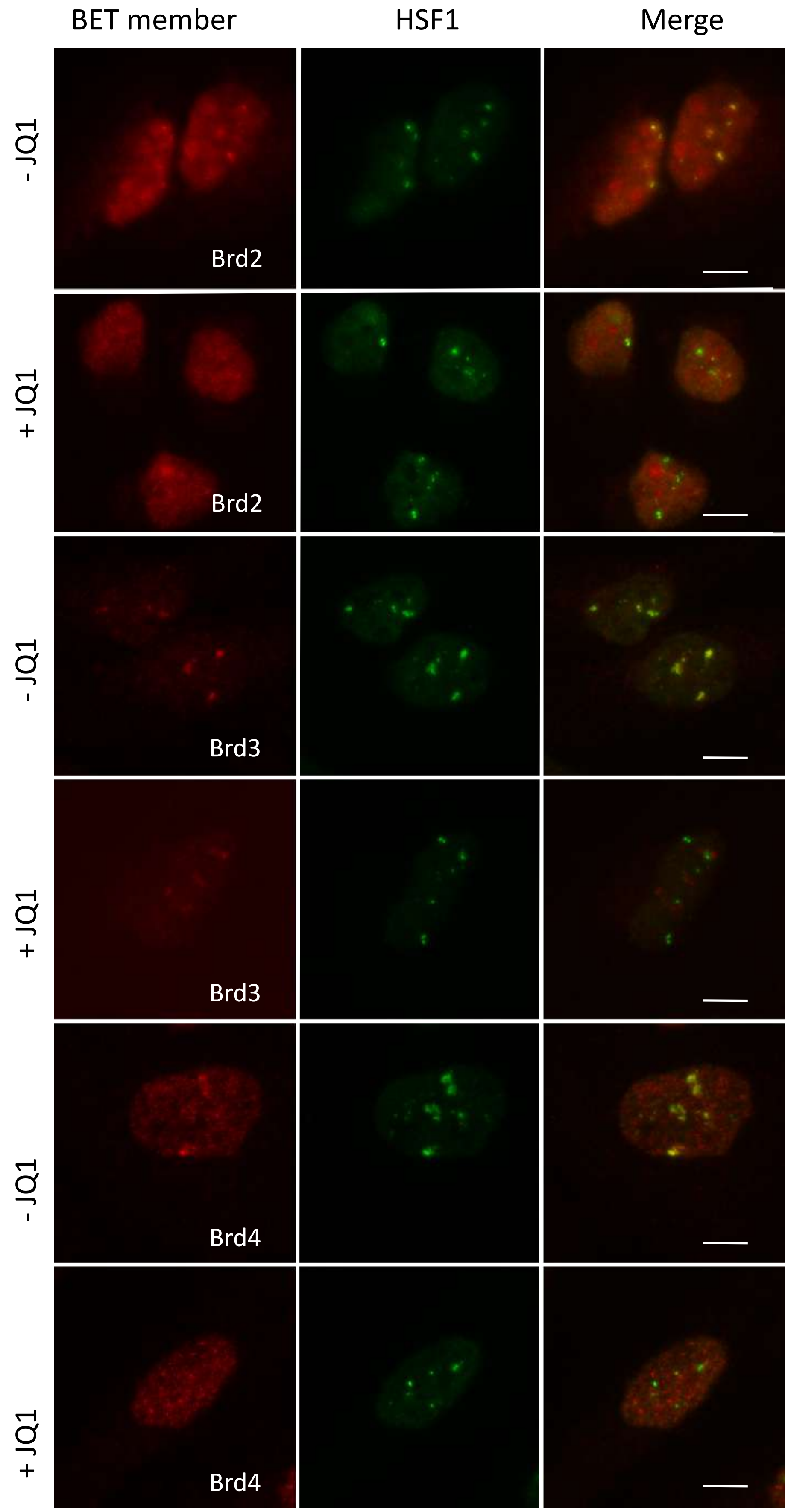

Supplementary Fig.S3

Detection of endogenous BRD2 and BRD3 and BRD4 by immunofluorescence inheat-shocked HeLa cells. Endogenous BRD2, BRD3 and BRD4 proteins (red signals) co-localize with HSF1 foci (green signals) in heat-shocked cells. No colocalization between HSF1 foci and endogenous BRD 2, BRD 3 and BRD4 is observed in heat-shocked cells treated with JQ1. (Bar = 5 µm)

Supplementary Fig.S4

A

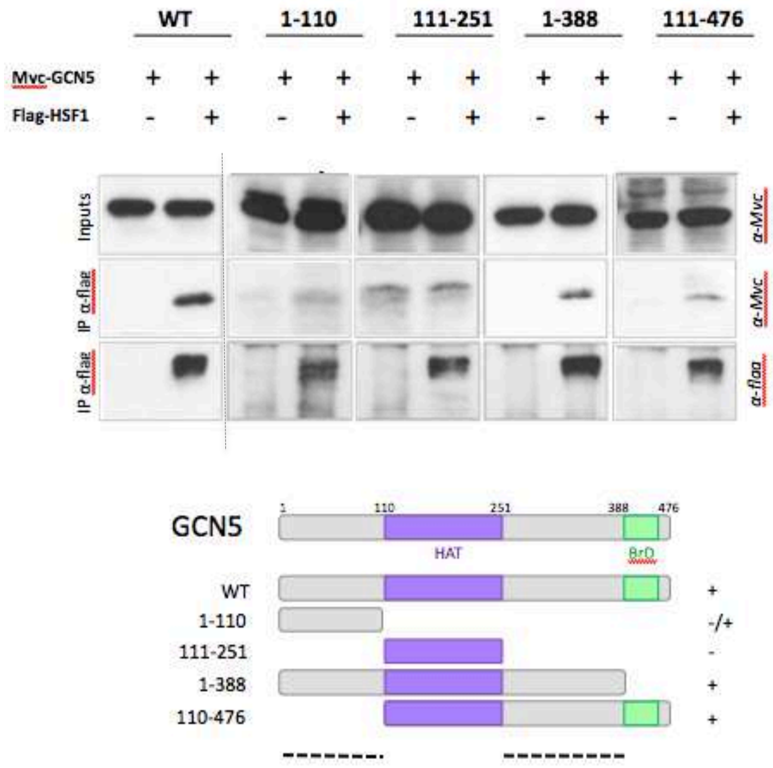

B

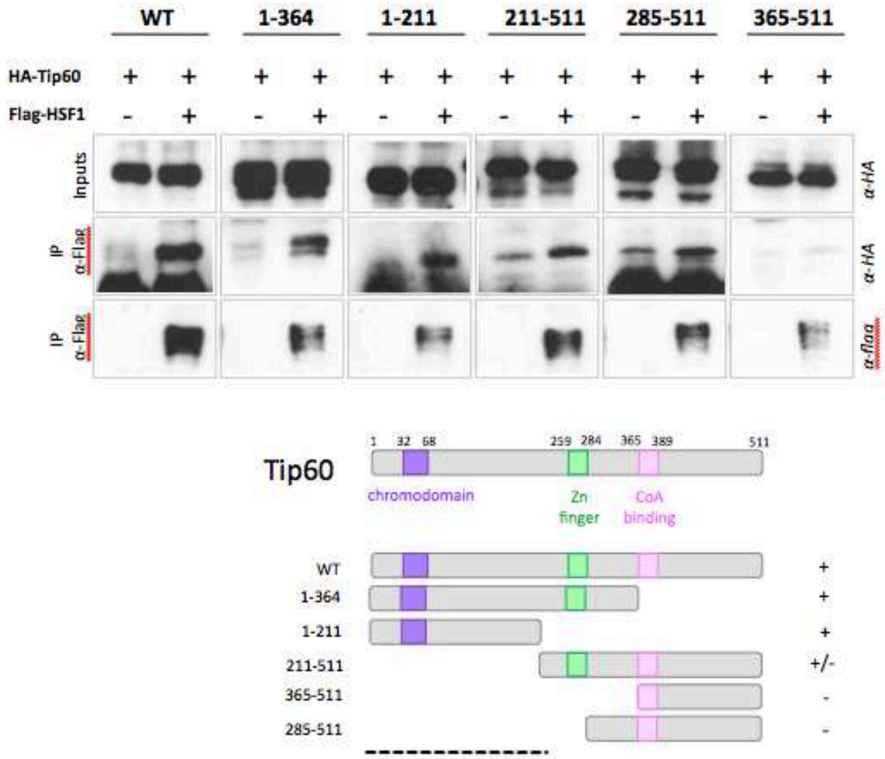

Supplementary Fig.S4

Mapping of Gcn5 and Tip60 domains involved in their direct or indirect interaction with HSF1.

Cos cells were transfected with a plasmid expressing Flag-HSF1 and a plasmid expressing wild type or mutated forms of Myc-Gcn5 or HA-Tip60. Immunoprecipitations were performed with an anti-Flag antibody. Gcn5 and Tip60 domains involved in the direct or indirect interaction with HSF1 are underlined. The dotted line delineates data obtained from two different gels.

Supplementary Fig.S5

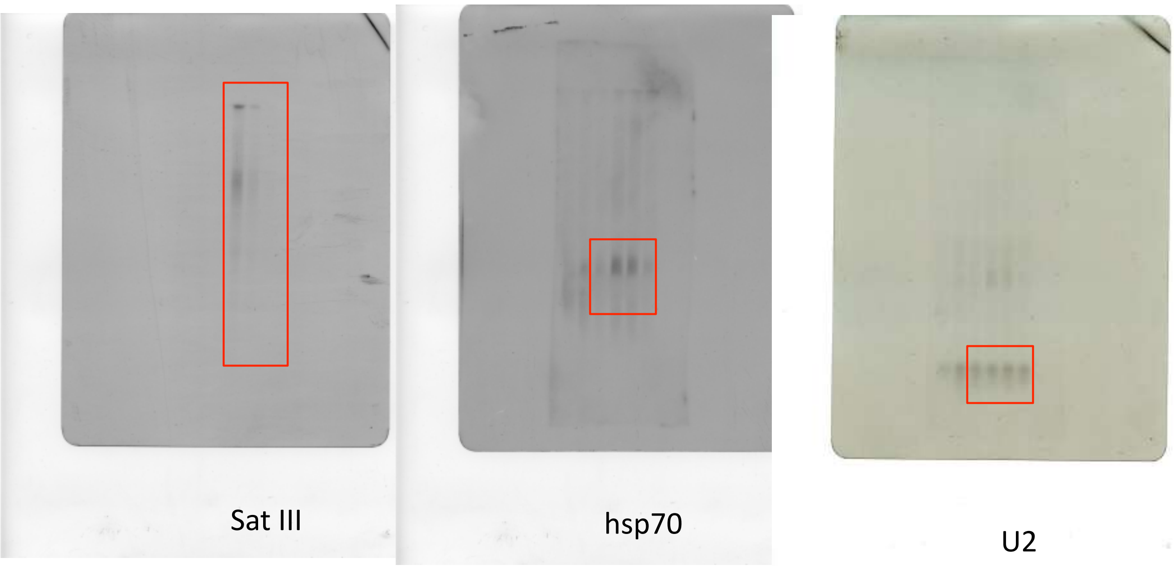

Original autoradiography images of the Northern blots presented in Figure 6c. The areas displayed in Figure 6 are outlined with red frames.
